# Does the Development of a High-Speed Railway Improve the Equalization of Medical and Health Services? Evidence from China

**DOI:** 10.3390/ijerph16091609

**Published:** 2019-05-08

**Authors:** Gangqiang Yang, Yuxi Ma, Yongyu Xue, Xia Meng

**Affiliations:** 1 Institute for the Development of Central China, Wuhan University, Wuhan 430072, China; gqyang@whu.edu.cn (G.Y.); mayuxi@whu.edu.cn (Y.M.); xueyongyu@whu.edu.cn (Y.X.); 2 Development Research Center of the Yangtze River Economic Belt, Wuhan University, Wuhan 430072, China; 3 School of Economics and Management, China University of Geosciences (Wuhan), Wuhan 430074, China

**Keywords:** HSR development, city, medical and health services, equalization level

## Abstract

Does the development of a high-speed railway (HSR) have a significant impact on the equalization of medical and health resources allocated among cities? Based on the panel data of 67 cities in China from 2007 to 2016, this paper investigates the direct and dynamic effects of HSR development on the equalization of medical and health services by using the difference-in-differences (DID) method. The empirical results show that an HSR connection significantly reduces the equalization level of medical and health services in cities and that the effect is larger for the period from the year of the connection to the second year. However, in the long term, HSR development improves the equalization level of medical and health services in cities. Heterogeneity tests show that the effect of the HSR connection shows an “N”-shaped trend under different city scales, the equalization level of medical resources in the largest cities benefit the most from HSR development, and the Eastern and Western regions of China are more sensitive to the HSR connection. While the allocation of medical resources is in the direction of equalization, the level of medical resources is significantly more equal with the HSR development in cities with stronger financial capacity and non-core cities. The analysis of other city characteristics provides policy recommendations for improving the public services delivery mode in China’s heterogeneous cities in terms of HSR development.

## 1. Introduction

As one of the basic elements of human production and life, transportation infrastructure is the link between socioeconomic activities and geographical space. The development of a high-speed railway (HSR) gives new meaning to transportation. In 1964, the Tokaido section of the “Shinkansen” in Japan, which has a speed of 210 km per hour, was opened to traffic, marking the birth of the first HSR in the traditional sense. Since then, HSR systems have been built and expanded in France, Germany, China, and other countries. In recent years, due to the rapid development of the HSR in China, the “Four Vertical and Four Horizontal” rapid passenger transport network has basically taken shape. China has devoted itself to building an HSR network with “Eight Vertical and Eight Horizontal” corridors based on this framework, regional connection lines, and supplementary intercity railways (In July 2016, with the approval of the State Council of China, the Ministry of Transport of China and the China National Railway Administration jointly issued the Medium and Long-Term Railway Network Plan (Adjusted in 2016), which was promulgated by the National Development and Reform Commission of China). By the end of 2018, the operating mileage of the HSR in China had exceeded two-thirds of the global mileage of the HSR, more than 29,000 kilometers, leading in transport density and the passenger traffic volume of the HSR service worldwide.

New Economic Geography Theory claims that the improvement in interregional transportation infrastructure will promote the spatial flow of economic factors and public resources, accelerating the accumulation of production and life elements to central cities and leading to aggregate effects [1,2,3]. The flow of elements will also promote economic diffusion from central cities to peripheral cities and lead to city integration and suburbanization [4]. The spatial effects of regional socioeconomic activities depend on factors such as market scope, transportation costs, and the interregional mobility of laborers [5], whose roles are enhanced by the optimization of transportation facilities and commuting efficiency [6].

Public service has significant externalities [7]. As an important transportation infrastructure, the HSR signifies that great progress has been made in public services, which also have spillover effects such as agglomeration and diffusion. On the one hand, the HSR has a positive spillover effect through regional integration that occurs due to the improved accessibility of transport hub cities in the country [8,9,10,11], which means that public resources can be shared and diffused within a certain distance to promote overall welfare [12,13]. The case study in Ortega et al. [14] based on the HSR in Spain shows evidence that regional accessibility increases as its coefficient of variation decreases, promoting regional equilibrium development. Case studies based in China show that the HSR has become an important engine for regional economic development, accelerating the urban–rural mobility of production elements and improving accessibility between core markets and regions with low economic activity, thereby narrowing the urban–rural income gap [15]. The development of the HSR effectively reduces the space-time distance between cities, weakens the dilution effect of increased spatial distance on positive spillover effects, and accelerates the effective allocation of resources by enhancing spatial flow and interregional sharing. The effect of city integration through the layout of the industry system brings about the centralization of workplaces and the suburbanization of residences as well as the sharing of medical services across cities. City integration breaks down regional administrative boundaries and provides new channels for the cross-border enjoyment of medical and health services.

On the other hand, the HSR has a negative spillover effect [16,17,18,19], which is more obvious in the agglomeration stage of regional economies. Traffic facilitation may be adversely affected by regional administrative divisions and market segmentation [20], changing the direction of the flow of interregional resources and the preferences of residents in terms of their public service choices. The case study based on the Rome–Naples HSR in Cascetta et al. [21] shows that HSR development promotes the re-election of residential areas according to the needs of different public resources so that the ability of families to access public services can be enhanced. In China, the provision of interregional health services is an important factor leading to accelerated population mobility [22]. However, the supply-and-demand mismatch in basic public services still exists due to rigid administrative divisions, which leads to a dilemma regarding the provision of basic public services in that they are "too crowded in large cities, of an insufficient scale in medium-sized cities, and are inadequate in small cities".

Provinces and municipalities in China are heterogeneous in terms of their degree of marketization and economic development. Under the household registration system (The household registration system in China refers to families should have an hukou (the proof of household registration) to live in urban areas. Otherwise, the availability of urban services and facilities will be limited. Residents with local household registration enjoy more rights in terms of education, labor insurance, social security, medical care, education, and unemployment protection than do permanent residents without local household registration. The household registration system has become a hindrance to the mobility of the labor force and the narrowing of the income gap among regions.), social security and welfare systems are divided into regions. There are significant differences in the provision of public services not only between urban and rural areas but also among regions, resulting in “nonequilibrium supply”. In China, medical and health services are generally supplied by local governments at all levels. The core issue studied by researchers in the field of public economics is defining the optimal decision regarding the structure of the provision of local public services. Public services have a spatial spillover effect that is measured by local fiscal expenditures [23,24]. However, local governments determine their total budget according to the registered household population. An increase in the non-household permanent resident population significantly reduces the relative level of local public welfare expenditures and creates a congestion effect [25]. Government competition due to fiscal decentralization has been proven to improve the efficiency of the supply of local public goods and to better meet the preferences of voters [26]. However, the Promotion Tournament Game (PTG) in China, which is a kind of promotion appraisal that uses GDP as the chief-indicator, has led to problems such as structural distortions in local fiscal expenditures and insufficient public expenditures for public services [27,28]. Due to the institutional background and various other reasons, it is difficult for public services to benefit the entire permanent population, resulting in "nonequilibrium demand". In the era of HSR, cities gradually lose their independence as they develop; the spillover of public services is strengthening, and the spatial agglomeration of the population is accelerating [29]. Regions with greater financial capacity and better medical resources have stronger cross-regional medical gravitation. The unbalanced structure of service provision and decreased efficiency in terms of the supply of public services are unavoidable and have become obstacles to the equalization of regional public services.

Few empirical studies have been conducted to examine the impact of HSR development on the equalization of local public services. Compared to other public services, medical and health services are less restricted by the current system (the China household registration) and are more important for the population. Compared with ordinary railway transportation, the HSR has a shorter travel time, a safer, more environmentally friendly performance, and it is more punctual. Compared with air passenger transport, the HSR is more flexible, more economical, and has more stations. Some studies have shown that the HSR has a substitution effect on short-distance air passenger transport [30,31]. Based on the motivation of pursuing higher-quality medical resources and reducing travel time, residents are more inclined to choose cities with more mature and higher-quality medical and health services. The original supply mechanism for medical and health services that considers the registered population is no longer suitable.

This paper first uses the difference-in-differences (DID) estimation method to test the direct effect of HSR connections and the dynamic effects of HSR development on the equalization of medical and health services in China. Second, based on data that quantifies heterogeneity in terms of city scale, political attributes, economic levels, and financial capacity, the grouping method and difference-in-difference-in-differences (DDD) model are used to analyze the different effects of the HSR. 

The remainder of this paper is organized as follows. The second section describes the data sources, measurement models, and variable selection. The third section describes the benchmark regressions and heterogeneity tests. The fourth section discusses the robustness of the empirical results, and the last section provides the conclusion.

## 2. Materials and Methods

### 2.1. Data Sources

**Figure 1:**
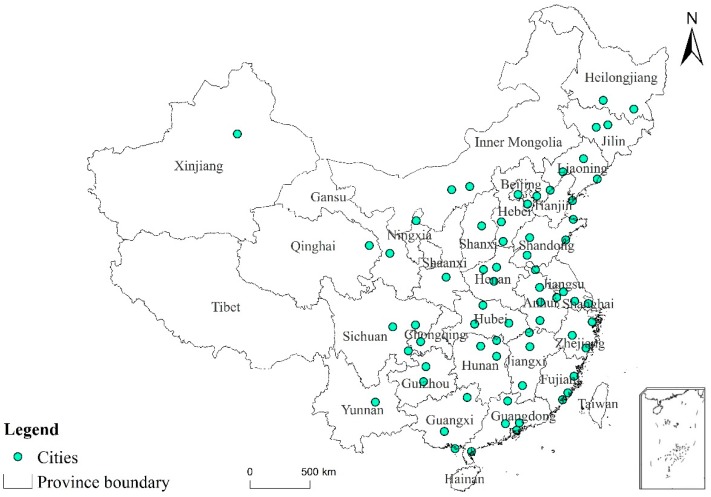
The location of the sample cities.

This paper uses the panel data of 67 cities in China from 2007 to 2016, covering all provinces and municipalities except Tibet, Taiwan, and Hainan, where there is no HSR line connected to the mainland of China. The relative location for the sample cities are shown by points in Figure 1. Some cities are located more closely together, in order to make the figure clearer we provide only the names of provinces in the figure.

For the empirical test, the HSR connection data were obtained from the professional inquiry platform of the “Gaotie.cn” (China High-speed Railway Website) (In this study, the “Gaotie.cn (http://www.gaotie.cn)” is regarded as an important, but not the only, source of data. The process of finding data on the “Gaotie.cn” is as follows. Firstly, we found the route map of the HSR in China up to the year 2018 through the "line" section in the top indicator bar of the website. Then, we confirmed the time when the station city connects the HSR, and whether all the station cities on the line connected HSR at a uniform time through checking the detailed introduction of each HSR line in the bottom section of the homepage. Besides this, we also used search engines to confirm information that is compiled from documents and news on official websites to verify the authenticity and reliability of data sources, such as the “12306 China Railway (https://www.12306.cn)” and the “People.cn (http://www.people.com.cn)”, etc.). This website provides timely information on the HSR network from which we can obtain updated information on the adjustment of the latest railway operating lines. The other statistics were derived from the “China Regional Economic Statistical Yearbook” of every year, the provincial statistical yearbooks, and the city statistical yearbooks and city statistical bulletins. Following the International Railway Union (UIC) and the Medium- and Long-Term Railway Network Plan promulgated by the National Development and Reform Commission of China [32], the definition of “HSR” in this paper covers passenger dedicated lines (PDLs), newly constructed HSRs with line speeds of up to 250 km/h, and upgraded lines with speeds up to 200 km/h, including the “G” prefix–high-speed Electric Multiple Units (EMU) passenger train, the “D” prefix–fast EMU passenger train, and the “C” prefix–intercity EMU passenger train.

### 2.2. Method

Based on the existing literature [33,34], we use panel data and embed the DID method in a two-way fixed-effects model to develop an empirical model that can effectively identify the net effect of exogenous shocks and control for the endogenous relationship between the HSR connections and the equalization of medical and health services in cities.

(1) $$Medi_{it}=\beta_{0}+\beta_{1}HSR_{it}+\beta_{2}Time_{it}+\alpha X_{it}+\gamma_{t}+\mu_{i}+\varepsilon_{it}$$

The above equation is the basic model used in this paper, where subscripts *i* and *t* represent the city and year, respectively. $Medi_{it}$ is the equalization level of medical and health services in city *i*. $HSR_{it}$ is the virtual variable for the HSR connection (In this study, the HSR connection timeline is set as follows. If the HSR was officially opened at the beginning or middle of the month, the year of the opening month is set as the current year for HSR connection. If the HSR opened at the end of the month, we extend the opening time by one month; if it is extended to the next year, we define the next year as the current year for the HSR connection. For example, the Wuhan–Guangzhou section of the Beijing–Guangzhou HSR was opened on December 26, 2009, so the year 2010 was set as the current year for the Wuhan–Guangzhou HSR connection.); if the year *t* is the current year for city *i* connecting the HSR for the first time and later for HSR development, the value is 1, and the value is 0 before city *i* connects to the HSR. $Time_{it}$ is the length of the developing period from the current year that city *i* connects to the HSR. $\beta_{2}$ represents the overall trend of the equalization of medical and health services as the number of years that the HSR has been developed for city *i* increases. The variable is used to determine whether the long-term development of the HSR can improve the equalization of medical and health services. $X_{it}$ denotes a vector of control variables. $\gamma_{t}$ and $\mu_{i}$ indicate individual-fixed effects and time-fixed effects, respectively. $\varepsilon_{it}$ is a random disturbance. In this model, the coefficient $\beta_{1}$ measures the direct effect of the HSR connection.

To test whether the HSR has a dynamic effect on the equalization level of medical and health services of cities, we set the two-way fixed-effects model as (2):

(2) $$\begin{array}{l} Medi_{it}=\beta_{0}+\theta_{1}after_{0}+\theta_{2}after_{1}+\theta_{3}after_{2}+\theta_{4}after_{3}+\theta_{5}after_{4}+\theta_{6}after_{\geq 5}\\ \;\;\;\;\;\;\;+\alpha X_{it}+\gamma_{t}+\mu_{i}+\varepsilon_{it} \end{array}$$

where $after_{j}$ (j = 0,1,…,4,≥5) is a virtual variable representing the dynamic effects of HSR. If $\theta_{1}$ is significantly negative in the regression, then the HSR connection has an instant impact on the equalization level of the medical and health services in city *i* in the current year. If the regression coefficients ($\theta_{1}\sim \theta_{6}$) are constantly statistically significant as *j* increases, then the HSR connection has a long-term effect. The other parameters have the same meanings as in Equation (1).

Finally, to further estimate the heterogeneity of the effect of the HSR connection, the variables representing the heterogeneity of city *i* are added to equation (1) to construct a DDD model (3).

(3) $$Medi_{it}=\beta_{0}+\beta_{1}HSR_{it}+\beta_{2}Time_{it}+\emptyset D_{it}\times HSR_{it}+\alpha X_{it}+\gamma_{t}+\mu_{i}+\varepsilon_{it}$$

$D_{it}$ represents the virtual variable that indicates the heterogeneous impact of the HSR on the equalization level of medical and health services. Specifically, we want to verify the impact of the HSR on medical services in four regions, Eastern, Central, Western, and Northeastern China (To reflect the level of social and economic development in the different regions in China and to provide a basis for formulating regional development policies, based on some national policy documents China is divided into four economic regions, the Eastern, Central, Western and Northeastern regions. In this paper, cities in the Eastern region include Beijing, Fuzhou, Guangzhou, Hangzhou, Huizhou, Jinan, Jinhua, Jining, Nanjing, Ningbo, Qingdao, Qinhuangdao, Quanzhou, Shanghai, Shaoguan, Shenzhen, Shijiazhuang, Tangshan, Tianjin, Wenzhou, Wuxi, Xiamen, Xuzhou, Yangzhou, Yantai, and Zhanjiang. Cities in the Central region include Anqing, Handan, Bengbu, Changde, Changsha, Ganzhou, Hefei, Jiujiang, Luoyang, Nanchang, Pingdingshan, Taiyuan, Wuhan, Xiangyang (named Xiangfan before 2011), Yichang, Yueyang, and Zhengzhou. Western cities include Baotou, Beihai, Chengdu, Chongqing, Guilin, Guiyang, Hohhot, Kunming, Lanzhou, Luzhou, Nanchong, Nanning, Urumqi, Xi’an, Xining, Yinchuan, and Zunyi. Cities in the Northeastern region include Changchun, Dalian, Dandong, Harbin, Jilin, Jinzhou, Mudanjiang, and Shenyang.). We also investigate whether the impact of the HSR varies with the population size, financial capacity, and economic capacity of the city. The specific meaning of $D_{it}$ and some heterogeneous definitions will be introduced in Section 3.3. The other parameters have the same meaning as in Equation (1).

### 2.3. Variables

#### 2.3.1. Explained Variable

In the measurement of the equalization level of regional public services, there are two perspectives, input and output. When considering the input perspective, scholars often choose indicators such as per capita financial budget and per capita expenditures to measure the equalization of public services [35]. When considering the output perspective, scholars mostly use the entropy method to construct a comprehensive evaluation system based on multiple public service projects [36]. In this article, the definition of equalization adopts the equalization of basic public services in the State Council’s “Notice of the State Council on Printing and Distributing the 13th Five-Year Plan to Promote Equalization of Basic Public Services” [37]. Equalization of basic public services refers to the basic rights of residents in the basic public service sector, and residents enjoy basic public services at roughly the same level, its core is to promote equality of opportunity. Equalization does not emphasize that all residents enjoy the same basic public services, rather, it is based on the premise of recognizing the differences between regions—urban and rural areas—and ensuring that residents enjoy basic public services above certain standards, the essence of which is “the equalization of the bottom line”.

In this article, we mainly consider the degree of equalization in quantity. Due to data limitations, we are unable to obtain complete and effective data on the quality of medical and health services at city level, which also provides the space for further research. In alignment with existing research [36], we used the entropy method to build a special index for the equalization of medical and health services considering the output perspective as the explained variable (*Medi*). The number of medical personnel and medical technicians (medical personnel refer to various doctors and nurses, and technicians include various technical staff) per 1000 permanent residents and the number of beds in medical institutions per 1000 permanent residents are selected as the original indicators to measure the equalization of medical and health services. The general calculation process of the entropy method is set as Equations (4)–(8) [38]. 

Let the statistical value of each indicator be $x_{ij}^{\prime }$, the subscript *i* means indicator while *j* is the city. To eliminate the influence of different units between indicators, the data are normalized by the extreme value method. The normalized matrix is $Z_{ij}$.

(4) $$Z_{ij}=\frac{x_{ij}^{\prime }-\underset{j}{\min}\left|x_{ij}^{\prime }\right|}{\underset{j}{\max}\left|x_{ij}^{\prime }\right|-\underset{j}{\min}\left|x_{ij}^{\prime }\right|}$$

Then, calculate the information entropy of each indicator, the information entropy of the *i*-th indicator is:

(5) $$e_{i}=-k\sum_{j}^{n}y_{ij}\ln y_{ij}\;(0<y_{ij}\leq 1)$$

(6) $$y_{ij}=\frac{Z_{ij}}{\sum_{j=1}^{n}Z_{ij}},k=\frac{1}{\ln n}\left(when\;y_{ij}=0,\;y_{ij}\ln y_{ij}=0\right)$$

After the $e_{i}$ is obtained, the entropy weight of the *i*-th index can be determined according to the following equation:

$$w_{i}=\frac{1-e_{i}}{\sum_{i=1}^{m}(1-e_{i})}\;(w_{i}\in [0,1]\;\sum_{j=1}^{n}w_{j}=1)$$

We can then obtain a single-index comprehensive score matrix, $S_{ij}$.

(8) $$S_{ij}=w_{i}\times Z_{ij}.\;$$

#### 2.3.2. Explanatory Variables

In this paper, we use three main explanatory variables. The core explanatory variable is the virtual variable representing the HSR. Virtual variables can capture the sudden transition of a city from "not connected to HSR" to "connected to HSR" and are widely used in related research [39,40,41]. The sample cities are divided into a treatment group and a control group according to the value of the $HSR_{it}$. The 54 cities with HSR connections during the period from 2007 to 2016 form the treatment group. The variable representing the length of time since the year of the HSR connection (Time) is the second explanatory variable in models (1) and (3), and the year of the HSR connection is used as the first year for Time. In the dynamic effects test, we mainly study the specific changes that occurred within five years and the general trend after five years, so we use $after_{j}$. as the third explanatory variable.

#### 2.3.3. Control Variables

Four kinds of control variables are used in this paper. The first category measures the development level of the cities [7] and consists of three sub-indexes as follows. The economic capacity is expressed by per capita Gross Domestic Product (Rgdp). The level of investment in medical and health services (Inves), measured by the number of medical institutions per 100 square kilometers, can eliminate the interference of the regional area on the equalization of health services. The gap between urban and rural development (Gap) is measured by the ratio of the per capita disposable income of urban residents to that of rural residents.

The second category is used to measure the finances of the local governments [42,43] and includes absolute indicators (Exp) and per capita indicators (Rexp) of medical and health expenditures. We also measure the decentralization of local finances (Stru), which draws on a new measurement method proposed by Chen [44] in combination with that proposed by Oates [45] and Lin and Liu [46], measured by the ratio of local net revenue to total local financial expenditures. The preference of the government to focus on medical and health services is measured by the ratio of medical and health expenditures to total fiscal expenditures (Expper) [47].

The third category measures the scale of the cities and population agglomeration. The attractiveness indicator (Migr) (The equation used to calculate the population inflow rate is the same as that used in the relevant yearbooks in China [22]. The calculation is as follows: the population inflow rate = (the number of permanent residents at the end of the year - the number of permanent residents at the end of last year - the number of permanent residents at the end of the last year × the natural growth rate of the population)/the number of permanent residents at the end of the year. If the population inflow rate of a city is positive, then the city has a net population inflow, and vice versa. If the rate is zero, then the population growth rate is consistent with the natural growth rate.) measures the yearly migration rate, and population density (Pop) measures changes in the scale of the city. The degree of population agglomeration is measured as the ratio of the urban permanent population to the total permanent population (Urban).

The fourth category measures traffic accessibility focusing on highways and air transport. Following studies on the impact of HSR connections on tourism [48,49,50], highway traffic mileage (Road) and a virtual variable representing airports (Airport) are used as control variables to better separate the population flow due to the HSR connection. 

Table 1 and Table 2 provide descriptive statistics for each variable. In Table 2, the significance levels are obtained through a paired sample t-test under a 95% confidence level. The higher the level of significance is, the greater the difference between the cities with HSR connections and those without. There are significant differences in most variables, so we cannot simply state that HSR connections have an impact on the equalization level of medical and health services in the city, which requires empirical testing based on controlling other variables.

## 3. Results

### 3.1. Total Equalization

Before conducting the empirical tests, we used the coefficient of variation (CV) to measure the difference in the average annual equalization of medical and health services (A-Medi) between the treatment group and the control group and to test whether HSR development has promoted the equalization of medical and health services of cities [51]. The calculation is as shown in Equation (9).

(9) CVA−Medi=σμ

σ and μ represent the standard deviation and mean value of annual medical services per 1,000 permanent residents. The smaller the CV is, the higher the equalization level of the medical and health services in this group.

**Figure 2:**
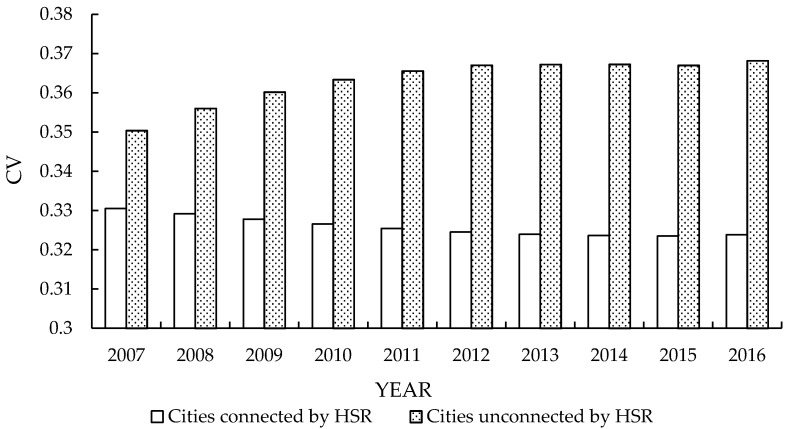
Average annual equalization of medical and health services.

Figure 2 indicates that the CV of the annual equalization level of the medical and health services of the treatment group shows a generally decreasing trend, while that of the control group is generally increasing. In conclusion, HSR development can effectively improve the level of medical and health services in cities in the long run. The following empirical model will be used for further analysis.

### 3.2. Benchmark Test

The test results for the direct effect of the HSR connection are reported in Table 3. The fixed effects (FE) were selected by using the Hausman test, and a collinearity test on the variables was performed, the result of which shows that the mean variance inflation factor (VIF) was 4.16, less than 10, indicating that the collinearity of the variables selected in this paper was not significant. Column FE (1) shows the regression results without the control variables. Columns FE (2)–FE (5) show the regression results with four kinds of control variables, namely, the comprehensive development level, the public finance level of the local governments, population agglomeration, and the accessibility of the cities.

The regression coefficient of the direct effect of the HSR connection and the positive effect of the length of time for HSR development on the equalization level of medical and health services are gradually strengthened, indicating that although the HSR connection has a negative impact, it will improve the equalization level of medical and health services and reduce the pressure of cross-regional medical services in the long run.

In the era of HSR, economic growth is conducive to improving the capacity of medical and health services. Rural residents have a more powerful motive to pursue urban medical and health services due to the HSR and the unbalanced allocation of medical resources between urban and rural areas, which increases the demand for urban medical and health services and reduces their overall level of equalization.

Highway construction, which is viewed in public services as an improvement in the infrastructure, reflects the comprehensive financial capacity of local governments, which has a positive relationship with the supply of medical and health services. The passenger flow brought by the operation of civil aviation lines has significantly reduced the equalization level of medical and health services, and the absolute value of the coefficient for the direct effect of the HSR increased significantly after controlling for highway mileage and civil aviation. This result shows that the improvement in long-distance and short-distance passenger transportation modes, represented by HSR and civil aviation respectively, can fully exert the influence of transportation infrastructure on regional public services. Therefore, controlling for the impact of other modes of transportation in the analysis of the HSR direct effect is correct and necessary.

To investigate which city characteristics can optimize the equalization level of medical and health services considering the development of the HSR, this paper further adopts the cross-term regression method, which multiplies the virtual variable HSR and the control variables that have positive effects on the equalization level of medical and health services, as shown in Table 3. The results of the optimization mechanism test are reported in Table 4, which reports regression results only for the cross-terms that are statistically significant.

The results show that the government and social capital can improve the supply side by investing more in the medical and health care field to alleviate the pressure brought about by an increase in the population. 

HSR development has removed regional barriers and increased the level of urbanization and the demand for medical and health services through population agglomeration; cost sharing may promote the supply of medical and health services. The urban variable did not significantly promote the equalization level of medical and health services in Table 3, but the regression coefficient of HSR × Urban is significant. This finding shows that the positive effect of urbanization on the equalization level of medical and health services can be achieved through development of the HSR.

In addition, when the financial autonomy of the local government is high, more information can be used to better meet the medical needs of residents within the fiscal jurisdiction. In Table 4, the Stru begins to significantly improve the equalization level of medical and health services, which shows that it is necessary to give full play to the financial capacity of local governments in the development of HSR.

We then decomposed the dynamic effect of HSR development time by year to assess whether the HSR effect turns from negative to positive after a certain number of years.

According to the regression results shown in Table 5, the pressure of medical and health services caused by the HSR is instantaneous and is strongest in the year after connection. By the fourth year after the HSR connection onward, HSR development has an insignificant but positive impact on the equalization of medical and health services in cities. The above results show that HSR connection elicits a certain degree of medical crowding over the short term, resulting in a reduction in the per capita public resources and a relatively low supply level of medical and health services. However, the congestion related to medical and health services gradually decreases over time. Overall, the regression results for the dynamic effects show that cities should focus on the rapid increase in the demand for medical and other public services from the year of the HSR connection to the second year after the HSR connection and should pay more attention to increasing the supply of medical and health services over the long term.

### 3.3. Heterogeneity Test

The above empirical analysis shows that HSR development has a considerable impact on the equalization of medical and health services between cities. Next, we explore the performance of the HSR effect considering heterogeneous city characteristics. First, we conduct a grouping test based on the heterogeneity of city scale and then perform a DDD heterogeneity test for cities with different political attributes and financial capacities, as well as their geographical position in different economic regions.

City scale represents the potential comprehensive development ability of the city. Some studies have reported that there is a statistically positive correlation between city scale and the capacity of the floating population, the level of public service, the floating population’s recognition of the region, and the willingness to settle or leave. All of these factors were shown to have an important impact on the change in city scale and its rank order [52]. It is recognized that an HSR is essential for improvement in the public services offered in a city, which can enhance the geographical identity of the floating population. In this study, city scale is measured by the permanent population size of urban areas. According to the official classification criteria (In 2014, the State Council of China issued the “Notice on Adjusting the Standards for Dividing Urban Sizes”, which took the permanent population of urban areas as the statistical caliber and divided the cities into five categories. Cities with an urban resident population of 200,000 to 500,000 are considered to be small cities, those with a population of 500,000 to 1,000,000 are medium-sized cities, those with a population of 1,000,000 to 5,000,000 are large cities, those with a population of 5,000,000 to 10,000,000 are megacities, and those with a population over 10,000,000 are super-large cities (the high numbers are included, while the low numbers are excluded).) used by the State Council of China, which divide cities into five categories, this paper divides the sample cities into four categories by combining cities with a population of 200,000–500,000 and cities with a population of 500,000–1,000,000. Then, a grouping test is conducted by separating regression per class of cities to analyze the direct effect of the HSR connection on the equalization level of medical and health services. The regression results are presented in Table 6.

**Figure 3:**
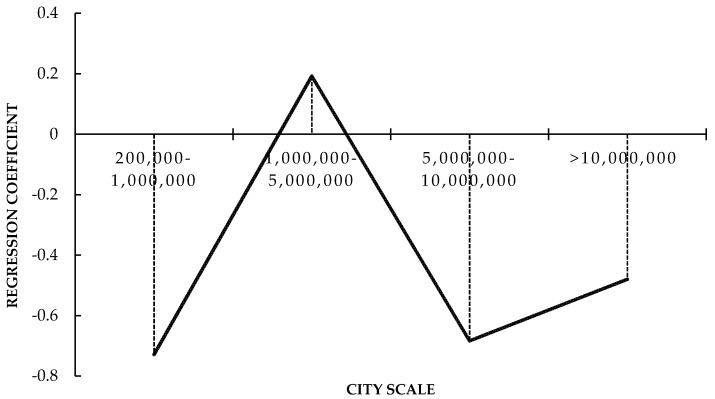
Trend of the regression coefficients for the direct effect with different city scales.

The regression results show that there are significant differences in the direct effect of HSR in cities with different population scales. The regression coefficients of the four categories of cities show an "N"-shaped change with the evolution of the city scale (Figure 3).

Cities with the smallest population scale are most sensitive to the effects of the HSR connection. This paper considers that these cities have more space and bearing capacity to carry inflowing populations that continue to migrate from rural areas and lower-level cities, so the increasing population density and degree of agglomeration lead to a decline in the equalization of medical and health services.

When the population scale reaches 1,000,000 to 5,000,000, due to the considerable pressure of increasing population density, cities begin to build new subcenter cities around the HSR station, which effectively diffuses the population, pushing them to the surrounding areas, thus reducing the pressure of the medical and health services offered by the central city. When cities are larger, the coefficient of the direct effect of HSR is significantly negative. Most provincial capital cities belong to this category, their unique political attributes attract the population in a sustained manner. Therefore, the population inflow caused by the HSR connection significantly reduces the equalization level of medical and health services. However, in cities with a population of more than 10,000,000, due to the highly mature operating system and city layout, medical and health services are relatively adequate, and the crowding effect caused by the inflow of migrants is eased. HSR development disperses the population and relieves population density, which subsequently alleviates the pressure of public goods in cities, so HSR development brings the most improvement effects.

**Figure 4:**
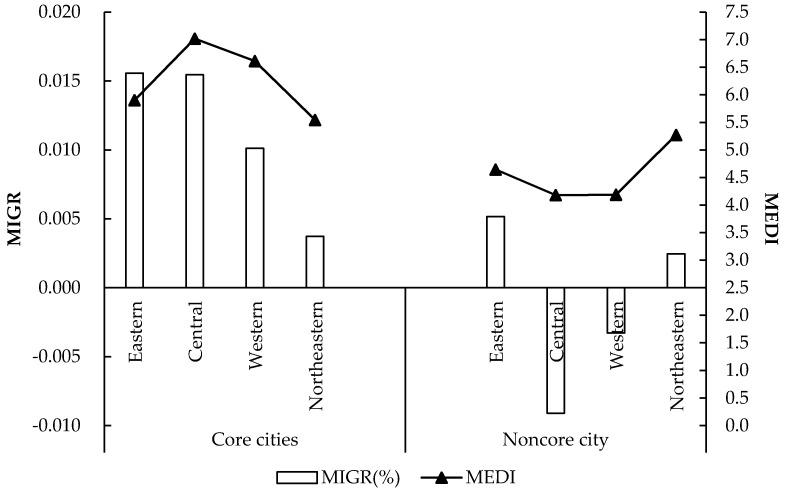
Population flow rate and the level of medical and health services in cities in different economic regions.

Next, we used the DDD model to test the heterogeneity of cities in terms of their political attributes, financial capabilities, and the economic regions in which they are located. The regression results of the effect of the HSR connection are shown in Table 7, the cross-term shown in the table means the $D_{it}$=1 in equation (3), and Table 8 shows the effect of the HSR development based on classification samples. To further analyze the direction of population flow, in Figure 4 we show the yearly migration rate of the population (Migr) in cities with different political attributes in four different regions.

For the heterogeneity test, we used the following definitions for “core city” and “noncore city”: the provincial capital cities and municipalities directly under the central government are considered to be core cities, and all other cities are classified as noncore cities. For noncore cities, an HSR connection has a strong negative effect, while the equalization level of medical and health services in core cities tends to significantly improve after the HSR connection. This result indicates that the inherent foundation of medical and health services offered in core cities and the relatively mature construction of subcenter cities along HSR lines substantially reduce the pressure that occurs due to population inflow. This process takes precedence over many noncore cities, so the equalization of medical resources in noncore cities depends more on the development of HSR.

The level of economic development in the Eastern region is relatively high compared to that in the other three regions and has been accompanied by a large population inflow. In this region, the increasing demand of medical resources has outpaced the increasing supply, the HSR connection effect is significant. In the Central region, we find that the equalization level of medical and health services is the highest among the four regions. The core cities are experiencing population inflow, and the noncore cities are experiencing net outflow. Therefore, although the equalization level of medical and health services after the HSR connection has declined, it is not significant. The HSR network in the Eastern and Central regions has developed rapidly, and the regional development has transitioned to the stage of city groups. Therefore, the allocation of medical and health resources is more dependent on the development of the HSR. 

Most of the core cities in the Western region have a population scale ranging from 200,000 to 5,000,000 with a positive population inflow rate, and the noncore cities have a population scale smaller than 1,000,000 with a net outflow rate. These noncore cities with HSR connections are all in the early stage of HSR development. They have been experiencing the stage of industrial agglomeration and undertaking the transfer of labor-intensive industries from the Central and Eastern regions caused by HSR development, causing a large number of rural people to migrate to urban areas. Therefore, the increase in the supply of medical and health public services cannot meet the rapid increase in demand, so the HSR connection is significantly negative. The Northeastern region is in a critical period of industrial transformation, moving from traditional secondary industries to advanced technical industries. The net population inflow indicates that human capital is being used to enhance advanced industries and sustainable development, so the population scale is growing even further, leading to the significantly continuous negative effect of HSR development. However, the Northeastern region is experiencing a high degree of population agglomeration and urbanization because it is becoming industrialized, and they are developing a more mature and equal system of medical and health services, which ranks second among the four regions. Therefore, although the growth of demand surpasses the increasing supply, the negative effect from the HSR connection is not significant.

In this paper, per capita medical and health expenditures are growing at a rate that is faster than or equal to the growth rate of per capita GDP in cities with considerable fiscal capacity, and vice versa. The medical and health services of cities with a stronger capacity are more sensitive to the effects of the HSR connection. This result shows that although medical expenditures are growing faster than economic growth, the more abundant and high-quality the medical resources are, the easier it is to attract people to enjoy medical and health services across regions. However, the reduction in equalization caused by the HSR connection in cities with weaker financial capacity is more obvious. Cities with stronger financial capacity depend more on HSR development to alleviate the crowding effect, and cities with weaker financial capacity should focus on improving their financial capacity first.

## 4. Discussion

In this section, we performed some auxiliary tests on the tests above to ensure the robustness of the conclusions of this paper. When using the DID method, if there is no external impact from the HSR connection, the development of the equalization level of medical and health care services in the treatment group and the control group should have the same trend over time, and there should not be any systematic differences. Because the HSR connects to different cities at different times, following the literature [53,54] the year 2009—in which the largest number of cities connected to the HSR for the first time—is selected as the time point that has an external impact for the parallel trend test, which helps us construct the virtual treatment group and the control group. The time trends of the equalization level of medical and health services were compared between the two groups to provide a visual explanation.

**Figure 5:**
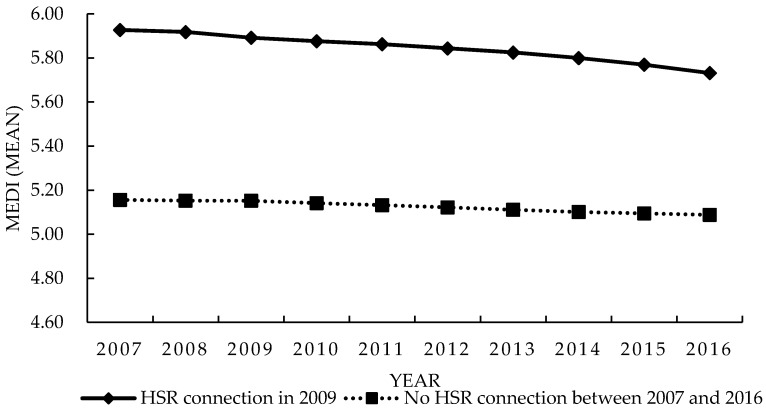
Parallel trend test of the HSR.

Figure 5 shows that before 2009, the equalization levels of medical and health services in the two groups were basically parallel, and there were no systematic differences over time. After 2009, the equalization level of medical and health services in the treatment group showed a clear downward trend. From 2009 to the end of the study period, the equalization level of medical and health services in the control group decreased by 1.245%, while that of the treatment group decreased by 2.795%, indicating that HSR connection plays a role in this change.

Next, we considered the study period up to and including 2009 and used the difference in the equalization level of the average annual medical and health services of both the treatment group and the control group as the explained variable, and the equalization level of the medical services in the treatment group as the explanatory variable to study the trend of equalization level of medical and health services for both groups. The results of ordinary linear regression show that the P value of the treatment group for the equalization level of medical and health services is 0.054 under the 95% confidence level, indicating that the equalization level of medical and health services of the two groups did not differ significantly before 2009.

To verify the hypothesis regarding the parallel trend, the counterfactual method was used to further evaluate the parallel trend [49,55]. We shifted the time of the HSR connection in each city one year, two years, and three years ahead of schedule and constructed the virtual variables before1, before2, and before3. If the coefficients of these variables are still significantly negative, indicating that the change in the equalization level of medical and health services for a city is affected by other random factors and is not entirely caused by the HSR connection, then the above test conclusions are not robust.

The regression results shown in Table 9 indicate that the coefficients of the three virtual variables before1, before2, and before3 are positive and not significant, explaining that before the HSR connection, the role of other factors in the city caused the medical services to show an overall trend of improvement by year. Therefore, the change in the equalization level of medical and health services is not caused by other factors but by the HSR connection in the cities. This finding further verifies the conclusions based on the results of the benchmark regression.

To address the systematic differences in the trends of the treatment group and the control group in terms of medical and health services and to reduce the estimation error inherent in the DID method, propensity score matching (PSM) was used to conduct an additional robustness test to determine whether there was a significant difference between the two groups after matching. In this paper, the weight is determined by using the kernel matching method, and individuals in the same value range are matched.

Rosenbaum and Rubin [56] proved that only the absolute value of the standard deviation after matching is less than 20%, which is used as a standard for the matching effect. The results of the balance test, which are reported in Table 10, indicate that the standard deviations of the matching variables of the two groups are less than 20% after kernel matching, so there is no significant difference in the covariates, proving that the PSM method can effectively reduce the bias in the DID method caused by differences in the trends. After conducting the balance test, we re-estimated the direct effect and dynamic effect of the HSR, and the results are shown in Table 11. The results indicate that adding the control variables did not affect the coefficient of the direct effect significantly.

The impact of the HSR connection on medical and health services remains significant from the year of the connection to the second year and is not significant thereafter. The HSR connection has long-term positive regression coefficients on the level of equalization of medical and health services. The results of the estimation after matching are consistent with the results of the benchmark estimation.

## 5. Conclusions

This empirical research shows that an HSR connection has a greater impact on the equalization level of medical and health services in terms of permanent residents in one city, but the HSR has a positive effect on the development process. Our heterogeneity analysis shows that the impact of the HSR connection on the equalization level of medical and health services is based on the joint effect of the economy, population, government, etc. In addition, we also conclude that cities with different characteristics rely on the HSR development in different ways to improve the equalization level of medical and health services. To minimize the negative impact of the HSR connection and to change the effect from negative to positive as quickly as possible, it is necessary to take effective measures to improve the equalization level of medical and health services.

First, local governments should give full play to their financial autonomy, rebuild the supply mechanism for public goods based on the number of permanent residents, increase expenditures for public services – especially in important public fields such as medical and healthcare and education, and promote fiscal equality at all levels of government [57]. The cooperation between local government and social capital should be strengthened by build a diversified management system to better meet the demand for medical and health services.

Second, cities should be aware of the “N”-shaped trend of medical and health services. Cities with a population of less than 1,000,000, which are mostly in the agglomeration stage, should increase fiscal expenditures to support industrial development while providing financial support to the public service sector and subsequently increasing the supply of public services. Cities with a population of 1,000,000 or more should rationally guide population distribution, balance the layout of medical resources, and accelerate the construction of new HSR subcenter cities to reduce crowding in the central cities.

Third, regional coordinated development should be planned, and the public service system should be improved. Economically developed regions should make the most of their economic advantages, actively guide the rational distribution of population inflows, and continually increase financial and social investment in the medical and health fields. Economically underdeveloped regions should actively carry out industrial development and transformation to build new growth engines, reduce excessive population outflows, financially support public services, and guide social capital to improve the construction of infrastructure. These recommendations should be made to improve the level of medical and health services in cities that have been affected by HSR connection and HSR development.

We hope to use spatial measurement to consider the accessibility of medical resources for residents so that we can further explore the impact of HSR on the equalization of quality of medical and health services of the city as well as extend the concept of equalization to intercity and even city groups (i.e., not only within the city). This represents the direction of our future work.

## Figures and Tables

**Table 1:** Summary statistics for the total sample.

All Data	Unit	Scale	Mean	Std Dev	Max	Min
Medi	—	—	5.326	1.794	14.383	1.648
HSR	—	—	0.476	0.499	1	0
Time	—	—	1.879	2.625	10	0
Rgdp	RMB	Natural logarithm	10.721	0.580	12.028	8.886
Exp	10,000 RMB	Natural logarithm	12.419	0.953	15.269	9.621
Rexp	RMB	Natural logarithm	6.034	0.683	8.776	4.141
Expper	%	—	0.076	0.047	0.720	0.011
Stru	%	—	0.651	0.215	1.541	0.138
Inves	Number/100 square km	—	26.109	24.760	198.147	0.312
Urban	%	—	0.568	0.170	0.896	0.174
Gap	—	—	2.463	0.471	4.023	1.296
Migr	%	—	0.005	0.031	0.283	−0.239
Pop	Permanent population/square km	Natural logarithm	6.138	0.902	8.693	2.261
Road	km	Natural logarithm	9.322	0.683	11.870	7.165
Airport	—	—	0.876	0.328	1	0

**Table 2:** Summary statistics by group.

By Group	Cities Connected by the HSR		Cities Not Connected to the HSR		*p* Value
	Mean	Std. Dev	Mean	Std. Dev	
Medi	5.401	1.698	4.893	1.736	0.016 **
HSR	0.590	0.492	0.000	0.000	0.000 ***
Time	2.000	2.515	0.000	0.000	0.000 ***
lnrgdp	10.800	0.505	10.294	1.062	0.000 ***
lnexp	12.512	0.965	12.032	0.771	0.000 ***
lnrexp	6.051	0.693	5.989	0.928	0.000 **
Expper	0.075	0.052	0.081	0.028	0.053
Stru	0.696	0.203	0.464	0.160	0.000 ***
Inves	26.010	18.874	15.610	18.427	0.000 ***
Urban	0.585	0.163	0.468	0.131	0.000 ***
Gap	2.438	0.418	2.687	0.495	0.006 ***
Migr	0.006	0.031	–0.003	0.035	0.319
lnpop	6.288	0.671	5.305	1.062	0.000 ***
lnroad	9.284	0.692	9.481	0.620	0.923
Airport	0.720	0.307	0.156	0.394	0.023 **

Note: ** and *** denote the 5% and 1% significance levels, respectively.

**Table 3:** Test of the direct effect of the HSR connection.

Variables	FE (1)	FE (2)	FE (3)	FE (4)	FE (5)
No control variables					
HSR	−0.371 **	−0.376 ***	−0.377 ***	−0.391 ***	−0.412 ***
	[0.1179]	[0.1051]	[0.1041]	[0.1060]	[0.1068]
Time	0.0702 *	0.0822 **	0.107 ***	0.110 ***	0.114 ***
	[0.0277]	[0.0269]	[0.0276]	[0.0279]	[0.0282]
Development level					
Lnrgdp		1.979 ***	1.801 ***	1.786 ***	2.121 ***
		[0.3268]	[0.3790]	[0.3840]	[0.4020]
Inves		0.0350 ***	0.0339 ***	0.0338 ***	0.0336 ***
		[0.0032]	[0.0031]	[0.0032]	[0.0032]
Gap		−0.496 **	−0.537 **	−0.540 **	−0.619 ***
		[0.1735]	[0.1749]	[0.1768]	[0.1766]
Public finance level					
Lnexp			−2.772 ***	−2.988 **	−3.243 ***
			[0.6707]	[0.9449]	[0.9612]
Expper			1.701	1.891	2.143
			[1.3484]	[1.3660]	[1.3603]
Lnrexp			2.359 ***	2.547 **	2.783 **
			[0.6431]	[0.9433]	[0.9606]
Stru			0.479	0.414	0.345
			[0.4080]	[0.4154]	[0.4131]
Population agglomeration level					
Urban				0.676	0.511
				[0.7663]	[0.7673]
Migr				−0.0117	0.347
				[1.0789]	[1.0831]
Lnpop				0.358	0.726
				[0.9031]	[0.9233]
Traffic condition					
Lnroad					0.321
					[0.1876]
Airport					−0.544 *
					[0.2425]
Cons	4.253 ***	−14.96 ***	6.109	5.305	−0.699
	[0.1020]	[3.4911]	[6.1759]	[6.5930]	[7.0268]
*N*	670	660	660	658	649
Adj. R^2^	0.4651	0.5888	0.5997	0.5962	0.6090

Note: Standard errors clustered at the city level are in parentheses; *, **, and *** denote the 10%, 5%, and 1% levels of significance, respectively. All of the results are estimated using the difference-in-differences (DID) method embedded in the fixed-effects panel data model.

**Table 4:** Test of the optimization mechanism for the HSR.

Variable	FE (1)	FE (2)	FE (3)
HSR	−1.149 ***	−1.445 ***	−1.153 ***
	[0.1247]	[0.3720]	[0.2644]
HSR × Inves	0.0352 ***		
	[0.0034]		
HSR × Urban		1.804 **	
		[0.6184]	
HSR × Stru			1.116 **
			[0.3652]
Cons	6.559	5.364	0.869
	[7.0025]	[7.2083]	[6.9918]
Control variables	yes	yes	yes
Time effect	yes	yes	yes
Individual effect	yes	yes	yes
*N*	649	649	649
Adj. R^2^	0.6068	0.6145	0.6149

Note: Standard errors clustered at the city level are in parentheses; *, **, and *** denote the 10%, 5%, and 1% levels of significance, respectively. All results are estimated using the fixed-effects panel data model.

**Table 5:** Test of the dynamic effects of HSR development.

Variables	FE (1)	FE (2)
after_0_	−0.132	−0.268 *
	[0.1193]	[0.1062]
after_1_	−0.317 *	−0.417 ***
	[0.1226]	[0.1083]
after_2_	−0.266 *	−0.302 *
	[0.1332]	[0.1190]
after_3_	−0.077	−0.089
	[0.1484]	[0.1309]
after_4_	0.066	0.066
	[0.1570]	[0.1401]
after_≥5_	0.134	0.087
	[0.1396]	[0.1317]
Cons	4.235 ***	−0.109
	[0.1025]	[7.0462]
Control variables	no	yes
Time effect	yes	yes
Individual effect	yes	yes
*N*	670	649
Adj. R^2^	0.4627	0.6087

Note: Standard errors clustered at the city level are in parentheses; *, **, and *** denote the 10%, 5%, and 1% levels of significance, respectively. All results are estimated using the fixed-effects panel data model.

**Table 6:** Test of the direct effect of the HSR considering the heterogeneity of city scale.

Variables	200,000–1,000,000	1,000,000–5,000,000	5,000,000–10,000,000	>10,000,000
HSR	−0.729 **	0.192	−0.684 ***	−0.480 **
	[0.2621]	[0.1516]	[0.1645]	[0.1740]
Time	0.276 ***	−0.0673	0.232 ***	0.306 ***
	[0.0741]	[0.0354]	[0.0468]	[0.0753]
Cons	−34.89	17.45 *	−9.103	14.95
	[21.7533]	[8.6089]	[12.1386]	[17.5231]
Control variables	yes	yes	yes	yes
Time effect	yes	yes	yes	yes
Individual effect	yes	yes	yes	yes
*N*	207	145	179	118
Adj. R^2^	0.6045	0.8238	0.7055	0.7897

Note: The name of the column is the city group with a population size of 200,000–1,000,000, 1,000,000–5,000,000, 5,000,000–10,000,000, and more than 10,000,000 (the high numbers are included while the low numbers are excluded). Standard errors clustered at the city level are in parentheses; *, **, and *** denote the 10%, 5%, and 1% levels of significance, respectively.

**Table 7:** Test of the effect of the HSR connection under different types of heterogeneity.

Variables	FE (1)	FE (2)	FE (3)
HSR	−0.599 ***	−0.439 **	−0.440 ***
	[0.1331]	[0.1498]	[0.1128]
Time	0.112 ***	0.101 ***	0.0898 **
	[0.0282]	[0.0290]	[0.0285]
HSR × Core city	0.387 *		
	[0.1656]		
HSR × Central region		−0.116	
		[0.1800]	
HSR × Western region		−0.587 **	
		[0.1872]	
HSR × Northeastern region		−0.540	
		[0.2763]	
HSR × stronger financial ability			−0.0319 **
			[0.0898]
Cons	2.370	−0.290	−3.778
	[7.1207]	[7.3276]	[3.5110]
Control variables	yes	yes	yes
Time effect	yes	yes	yes
Individual effect	yes	yes	yes
*N*	649	649	585
Adj. R^2^	0.6121	0.6100	0.5013

Note: Standard errors clustered at the city level are in parentheses; *, **, and *** denote the 10%, 5%, and 1% levels of significance, respectively. All results are estimated using the fixed-effects panel data model.

**Table 8:** Test of the effect of the HSR development under different types of heterogeneity.

	Political Attributes		Economic Regions				Financial Abilities	
	Core	Noncore	Eastern	Central	Western	Northeastern	Weaker	Stronger
Time	0.0536	0.165 ***	0.292 ***	0.205 ***	0.0745	−0.324 **	0.0237	0.112 ***
	[0.0320]	[0.0448]	[0.0672]	[0.0363]	[0.0753]	[0.0997]	[0.0323]	[0.0659]
Cons	16.77 *	−2.723	11.80	−7.273	50.66 ***	12.41	−0.580	7.897
	[8.1639]	[11.9400]	[18.4440]	[8.7476]	[14.1636]	[28.8711]	[3.7923]	[6.1672]
Control variables	yes	yes	yes	yes	yes	yes	yes	yes
*N*	285	364	249	160	166	74	478	107
Adj. R^2^	0.7760	0.5459	0.5648	0.8553	0.7085	0.6481	0.5101	0.7307

Note: Standard errors clustered at the city level are in parentheses; *, **, and *** denote the 10%, 5%, and 1% levels of significance, respectively. All the results are estimated using the DID method embedded in the fixed-effects panel data model. The virtual variable HSR is concluded.

**Table 9:** Test of the common trend assumption, using a counterfactual test.

Variables	FE (1)	FE (2)
before1	−0.084	0.00605
	[0.1317]	[0.1300]
before2	−0.085	0.0610
	[0.1392]	[0.1315]
before3	0.057	0.154
	[0.1573]	[0.1401]
Cons	4.243 ***	0.384
	[0.1083]	[7.1243]
Control variables	No	yes
Time effect	Yes	yes
Individual effect	Yes	yes
*N*	670	649
Adj. R^2^	0.4525	0.5981

Note: Standard errors clustered at the city level are in parentheses; *** denotes the 1% level of significance. *N* is the number of observations. All results are estimated using the fixed-effects panel data model.

**Table 10:** Balance test of the PSM.

Variables	Mean		Bias (%)	t	*p* > |t|
	Treatment Group	Control Group			
Lnrgdp	10.975	10.949	5.5	0.77	0.443
Inves	30.845	29.967	4.8	0.54	0.591
Gap	2.354	2.324	7.3	0.98	0.327
Lnexp	12.787	12.673	14.1	1.84	0.066
Expper	0.080	0.074	13.3	1.31	0.190
Lnrexp	6.289	6.209	13.4	1.87	0.062
Stru	0.715	0.728	−6.9	−0.81	0.421
Urban	0.603	0.607	−2.8	−0.30	0.762
Migr	0.008	0.006	5.1	0.61	0.543
Lnpop	6.377	6.473	−12.6	−1.77	0.078
Lnroad	9.424	9.299	19.4	2.30	0.022

**Table 11:** Test of the direct and dynamic effects of the HSR after matching.

Variables	FE (1)	FE (2)
HSR	−0.411 ***	
	[0.1154]	
Time	0.122 ***	
	[0.0314]	
after_0_		−0.273 *
		[0.1096]
after_1_		−0.436 ***
		[0.1123]
after_2_		−0.331 **
		[0.1253]
after_3_		−0.085
		[0.1379]
after_4_		0.100
		[0.1535]
after_≥5_		0.026
		[0.1482]
Control variables	yes	yes
Time effect	yes	yes
Individual effect	yes	yes
Cons	−8.340	−8.042
	[7.8054]	[7.8272]
*N*	572	572
Adj. R^2^	0.5770	0.5776

Note: Standard errors clustered at the city level are in parentheses; *, **, and *** denote the 10%, 5%, and 1% levels of significance, respectively. All results are estimated using the fixed-effects panel data model.
